# HERV-K(HML7) Integrations in the Human Genome: Comprehensive Characterization and Comparative Analysis in Non-Human Primates

**DOI:** 10.3390/biology10050439

**Published:** 2021-05-14

**Authors:** Nicole Grandi, Maria Paola Pisano, Eleonora Pessiu, Sante Scognamiglio, Enzo Tramontano

**Affiliations:** 1 Laboratory of Molecular Virology, Department of Life and Environmental Sciences, University of Cagliari, 09042 Monserrato, Cagliari, Italy; mp.pisano@unica.it (M.P.P.); e.pessiu@studenti.unica.it (E.P.); s.scognamiglio@studenti.unica.it (S.S.); tramon@unica.it (E.T.); 2 Istituto di Ricerca Genetica e Biomedica, Consiglio Nazionale delle Ricerche (CNR), 09042 Monserrato, Cagliari, Italy

**Keywords:** HERV, HERV-K, HML7, endogenous retroviruses, retrotransposons

## Abstract

**Simple Summary:**

The human genome is not human at all, but it includes a multitude of sequences inherited from ancient viral infections that affected primates’ germ line. These elements can be seen as the fossils of now-extinct retroviruses, and are called Human Endogenous Retroviruses (HERVs). View as “junk DNA” for a long time, HERVs constitute 4 times the amount of DNA needed to produce all cellular proteins, and growing evidence indicates their crucial role in primate brain evolution, placenta development, and innate immunity shaping. HERVs are also intensively studied for a pathological role, even if the incomplete knowledge about their exact number and genomic position has thus far prevented any causal association. Among possible relevant HERVs, the HERV-K supergroup is of particular interest, including some of the oldest (HML5) as well as youngest (HML2) integrations. Among HERV-Ks, the HML7 group still lack a detailed description, and the present work thus aimed to identify and characterize all HML7 elements in the human genome. Results showed that the HML7 group includes 23 elements and an additional 160 “scars” of past infection that invaded in primates mostly between 20 and 30 million years ago, providing an exhaustive background to study their impact on human pathophysiology.

**Abstract:**

Endogenous Retroviruses (ERVs) are ancient relics of infections that affected the primate germ line and constitute about 8% of our genome. Growing evidence indicates that ERVs had a major role in vertebrate evolution, being occasionally domesticated by the host physiology. In addition, human ERV (HERV) expression is highly investigated for a possible pathological role, even if no clear associations have been reported yet. In fact, on the one side, the study of HERV expression in high-throughput data is a powerful and promising tool to assess their actual dysregulation in diseased conditions; but, on the other side, the poor knowledge about the various HERV group genomic diversity and individual members somehow prevented the association between specific HERV loci and a given molecular mechanism of pathogenesis. The present study is focused on the HERV-K(HML7) group that—differently from the other HERV-K members—still remains poorly characterized. Starting from an initial identification performed with the software RetroTector, we collected 23 HML7 proviral insertions and about 160 HML7 solitary LTRs that were analyzed in terms of genomic distribution, revealing a significant enrichment in chromosome X and the frequent localization within human gene introns as well as in pericentromeric and centromeric regions. Phylogenetic analyses showed that HML7 members form a monophyletic group, which based on age estimation and comparative localization in non-human primates had its major diffusion between 20 and 30 million years ago. Structural characterization revealed that besides 3 complete HML7 proviruses, the other group members shared a highly defective structure that, however, still presents recognizable functional domains, making it worth further investigation in the human population to assess the presence of residual coding potential.

## 1. Introduction

The sequencing of the human genome revealed that more than one half of it is constituted by transposable elements, among which LTR-retrotransposons—also called Human Endogenous Retroviruses (HERVs)—make up ~8% of our genetic material. Far from being only “junk DNA,” HERV sequences are progressively revealing their contribution to the host physiology. Besides the known cooption of (H)ERV envelope proteins to serve placenta development and homeostasis in eutherian mammals [1,2,3,4], growing evidence indicates that HERV integrations are important drivers of genomic innovation and had a pivotal role in the evolution and shaping of entire transcriptional networks, including major innate immunity pathways [5,6,7]. Such an impact on the host biology prompted also the investigation of HERV contribution to diverse diseased conditions, trying to link the various HERV groups’ expression to human pathogenesis. However, to date, no clear association between the expression of specific HERV loci and disease development has been established, and this is in part due to an incomplete knowledge of HERV abundance and diversity at the genomic level, which prevents the study of individual HERV loci expression and its effects on the host [8,9]. In fact, HERV cataloguing is still ongoing and presents some confusion, especially due to the use of a variable nomenclature and classification criteria not always based on precise taxonomical rules [10]. One of the most recent attempts of global classification has been performed with the software RetroTector, which scans vertebrate genomes searching for conserved retroviral motifs that are then evaluated to reconstruct the individual proviral insertions [11]. The latter are subsequently classified through a multiple approach based on taxonomical relationships and taking into account proviral integrity and composition by similarity image analysis [11]. In particular, RetroTector exploration of the human genome assembly GRCh37/hg19 led to the identification of 31 canonical HERV groups plus additional 39 non-canonical ones that revealed high degrees of mosaicism due to recombination or secondary integration events [12]. Importantly, such RetroTector-based classification can be used as a starting point for the deep characterization of individual HERV group of interest, which can possibly include additional members previously missed due to their structural incompleteness or accumulation of mutations affecting key retroviral motifs. In fact, while some HERV groups have been described in great detail (see for instance HERV-W [13,14] and HERV-H [15]), most of them are still poorly known in terms of total members, genomic distribution, and nucleotide structure. Overall, the best characterized class of HERV is surely Class II, which includes betaretrovirus-like elements currently divided into 10 groups named HML (Human MMTV-like) from 1 to 10 due to their identity to exogenous Mouse Mammary Tumor Virus (MMTV). Among HML groups, the best-described one is HML2, for which a full genomic characterization has been provided [16], revealing a prolonged activity in primates that led even to human-specific integrations polymorphic in the different populations [17]. Such an accurate knowledge of HML2 members supported the study of their possible role in a number of human pathologies, including various cancers and neurological diseases [18], making this group the most investigated in human disorders. Similarly, dedicated works of classification have been performed for HML1 [19], HML3 [20], HML4 [21], HML5 [22], HML6 [23], HML9 [19], and HML10 [24,25] groups. Contrarily, HML7 and HML8 elements still remain poorly characterized. In particular, the only data regarding HML7 elements come from a recent study investigating the overall differential expression of repetitive elements in the transcriptome of patients with lung cancer for biomarker identification purposes [26]. In the study, participants were divided into patients who developed lung adenocarcinoma (LUAD) and patients who have developed small cell lung cancer (SCLC). The analysis showed a significant upregulation of the inner portion of HML-7 elements (known as HERVK11D-int) specific to LUAD but not found in SCLC patients [26]. Importantly, the study evaluated the whole group modulation, i.e., without any information about the individual HML7 member expression. Aside from this result, no other information is available regarding the HML7 group composition and diffusion to date, and this prevents an exhaustive understanding of its single members’ expression in both healthy and diseased tissues. The present study has hence aimed for the comprehensive classification of the HML7 integrations present in the human genome, analyzing their context of insertion, proviral structure, and phylogenetic composition. In addition, the combination of time of integration estimation and comparative identification in the genome of non-human primates allowed us to provide a precise picture of the group dynamics of colonization of the primate lineages. Overall, results have finally clarified the composition of this hitherto poorly known HERV-K group, providing an exhaustive background for subsequent functional studies to assess their coding potential and the impact of individual HML7 loci expression on the host biology.

## 2. Materials and Methods

### 2.1. Identification of HML7 Loci and Solitary LTRs in Human Genome Assembly GRCh38/hg38

To collect the HML7 proviral integrations present into the human genome, we started from the 14 HML7 loci already identified in our previous work of global classification, performed with the software RetroTector [11] and aimed to the identification of the most intact HERV loci in the human genome [12]. To confirm their localization in hg38 genome assembly and identify eventual other HML7 loci, we performed a BLAT search in UCSC Genome Browser [27], using as a query the HML7 proviral reference MER11D-HERVK11D-MER11D as assembled from the Dfam database [28]. The identified genomic positions were then compared to RepeatMasker annotations and downloaded with 5′ and 3′ flanking sequences of 500 nucleotides each for the alignment with respect to the proviral reference, to assure their completeness for subsequent analyses. Similarly, HML7 solitary LTRs have been identified through BLAT searches in Genome Browser hg38 assembly with MER11D LTR reference and collected after the exclusion of the LTRs known to be associated to the proviral sequences identified in the previous step. In this case, the presence of the internal proviral portion has also been excluded through the download of sequences with 5′ and 3′ flankings of 500 nucleotides each, and their alignment with respect to the reference. All alignments were performed with MAFFT algorithms FFT-NS-I x1000 and G-INS-i [29] on Geneious Prime software, version 2020.1.1 (Biomatters Ltd., Auckland, New Zealand).

### 2.2. Genomic Distribution and Context of Integration

Once identified, we estimated the expected distribution of the HML-7 loci in each chromosome using the formula

*e* = *Cl* ∗ *n*/*Tl* (1)

where *e* represents the number of integrations expected in the chromosome, *Cl* is the length of the chromosome, *n* is the total number of HML7 loci in the human genome, and *Tl* is the sum of all chromosomes’ length. Considering the relatively low number of HML-7 proviruses (*n* = 23), the analysis was conducted taking into account also the integrations that have later been converted into solitary LTRs (*n* = 160). The variation of the expected integrations as compared to the observed ones was calculated for each chromosome with the chi-square test, and its statistical significance was estimated through the *p*-value.

We also assessed each HML7 locus context of integration, identifying the proviral sequences that were colocalized with cellular genes by intersecting their coordinates with the annotations of Gencode [30]. The obtained results were then visually inspected on Genome Browser, to evaluate the position of the HERV sequences with respect to the colocalized gene’s introns and exons.

### 2.3. Structural Characterization

HML7 loci have been aligned to the proviral reference sequence MER11D-HERVK11D-MER11D to annotate the position of LTRs and retroviral genes and to evaluate their integrity. Alignments have been performed and analyzed on the Geneious platform, as described above. All insertions and deletions with respect to the reference have been annotated, and the individual HML7 loci coding potential and functional domains have been evaluated using the software RetroTector [11] and NCBI Conserved Domains tool [31].

### 2.4. Phylogenetic Analyses

All phylogenetic analyses were done with MEGA-X software, version 10.1 [32,33]. Maximum likelihood trees were built using the Kimura 2-parameter model, and phylogenies have been statistically tested using the bootstrap method with 100 replicates. Neighbor joining trees were built with p-distance method applying the pairwise deletion option, and phylogenies were tested with 1000 bootstrap repetitions. In addition to the HML7 sequences found in the human genome and the group reference sequence as assembled from Dfam (MER11D-HERVK11D-MER11D), the alignment used to generate the trees included the Dfam references of the other HML groups (HML1 to 10). Alignments have been performed with MAFFT algorithms FFT-NS-I x1000 and G-INS-i [29] on Geneious Prime software, version 2020.1.1 (Biomatters Ltd., Auckland, New Zealand), and were visually inspected prior to phylogenetic analyses. The extraction of proviral subportions (e.g., to generate the trees of LTRs or individual retroviral genes) was made with Geneious software, taking into account nucleotide coordinates indicated by Dfam for each viral gene in HERVK11D reference: gag (~162–2027), pro (~1985–3032), pol (~3113–5590), env (~5804–7750).

### 2.5. Estimation of HML7 Loci Time of Integration

The integration time of HML7 elements has been estimated using the formula

*T* = *D*/0.2% (2)

which considers the coevolution of HML7 loci with the host DNA, applying a spontaneous substitution rate equal to the one of the human genome (0.2% mutations per nucleotide per million years) to the percentage of divergent nucleotides (*D*) of each HML7 locus with respect to a reference (that represents ideally the ancestral sequence). In particular, the divergence has been calculated: (1) for the nucleotide sequences of individual retroviral genes and LTRs with respect to a consensus sequence generated by the alignment of all members of the group, and (2) between the two LTRs of the same provirus, which are identical at the time of integration and then accumulate mutations according to the host genome substitution rate. In the latter case, the obtained *T* values were then divided by 2, considering that each LTR of the same provirus accumulated mutations independently after the integration. The divergence values were estimated with MEGA-X [32], using the pairwise deletion option and excluding Cpg dinucleotides, known to be subjected to hypermutation. The final estimated age of HML7 sequences was expressed as the average value of those obtained with the different approaches, excluding values with a standard deviation >20%.

To confirm the results obtained, the presence of each HML7 sequence was assessed in non-human primates’ genome assemblies through comparative genomic annotations available on UCSC Genome Browser, in order to identify the oldest common ancestor (O.C.A). In particular, starting from the coordinates in the human genome, each HML7 locus has been searched in primate species from both Catarrhini (chimpanzee, gorilla, orangutan, gibbon, rhesus macaque) and Platyrrhini (marmoset, squirrel monkey) parvorders, using the genomic flanking sequences to assure the actual correspondence.

## 3. Results

### 3.1. Identification of HML7 Loci in Human Genome Assembly GRCh38/hg38

The BLAT search conducted in UCSC Genome Browser [24] human genome assembly 38 (hg38) using the HML7 Dfam reference MER11D-HERVK11D-MER11D as a query led to the coordinates of various hits, which were extracted in FASTA format through the Table Browser tool, including 5′ and 3′ flanking sequences of 500 nucleotides each. The obtained sequences were then compared by multiple alignments to the reference, to exclude elements with a low level of identity (<90%) and to assure their completeness for subsequent analyses. Using this methodology, we identified a total of 23 HML7 sequences, 14 of which were originally included in our first work of HERV global classification, as performed with the software RetroTector [12] (Table 1). Each HML7 element has been designated with a unique name, corresponding to the genomic locus of insertion (Table 1). In addition, an analogous BLAT search using as a query MER11D LTR has allowed to detect about 160 HML7 solitary LTRs, resulting from the recombination between the two LTR of the same provirus, and the consequent removal of the inner portion (Supplementary File S1).

### 3.2. HML7 Loci Are Not Randomly Distributed among Human Chromosomes

**Figure 1:**
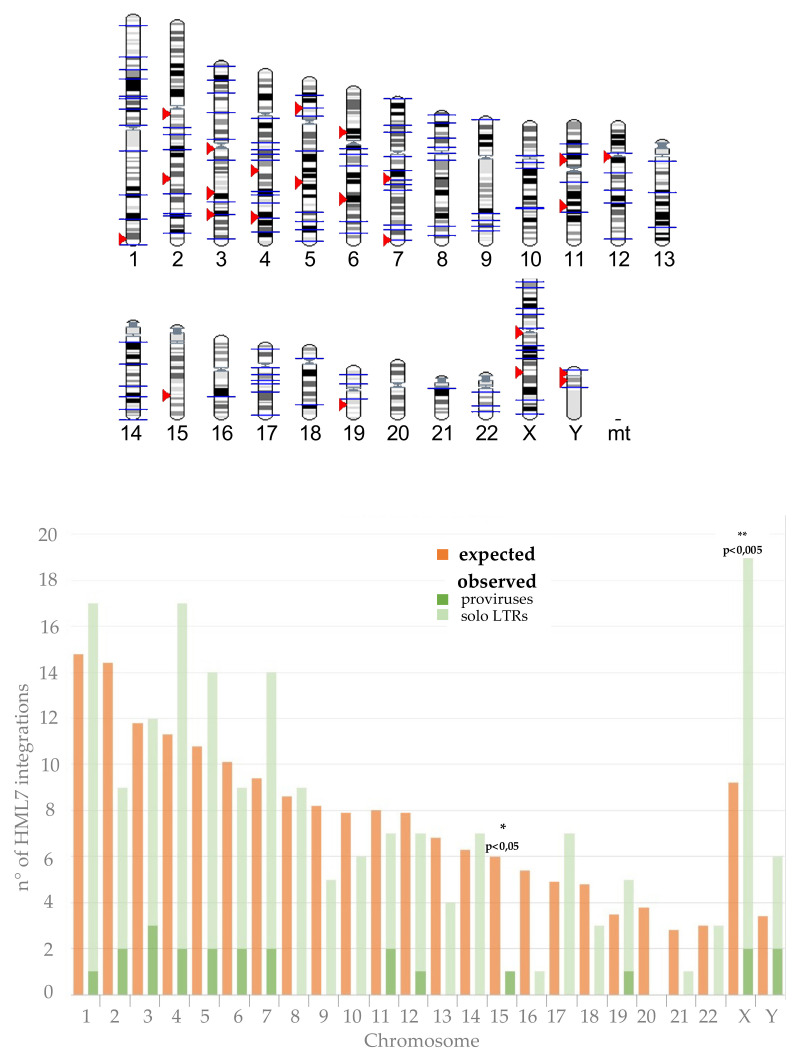
Chromosomal distribution of HML7 loci. In the upper part of the figure, HML7 proviruses (red arrows) and solitary LTRs (blue lines) have been visualized on the human karyotype (source: www.ensembl.org (accessed on 30 March 2021)). In the lower part of the figure, the observed chromosomal distribution of HML7 elements was statistically compared to the expected one, showing significant decrease in chromosome 15 and enrichment in chromosome X integrations.

Once identified, we assessed whether the HML7 integrations present in the human genome are randomly distributed in the various chromosomes or show instead some biases as compared to the expected frequency. To this purpose, given the relatively low number of HML7 intact proviruses, we also took into account the ancestral integrations that have undergone LTR-LTR recombination, and hence found currently as solitary LTRs. For each chromosome, we conducted a chi-square test comparing the observed number of HML7 integrations with the expected one. Briefly, the number of expected integrations in each chromosome has been obtained considering its length, the total number of HML7 loci in the human genome and the sum of all human chromosomes’ length. This number was then compared to the actual number of HML7 integrations through the chi-square test, and the statistical significance of the observed variation was tested through *p*-value calculation (Figure 1). Results showed a significant enrichment of HML7 insertions in chromosome X (where the expected insertions were 9 while the actual ones include 17 solitary LTR plus 2 proviruses); while chromosome 15 held fewer HML7 sequences than the 6 expected (only 1 provirus and no solitary LTRs) (*p* < 0.005 and *p* < 0.05, respectively) (Figure 1). In addition, chromosome 20 showed no HML7 elements, neither proviruses nor solitary LTRs (Figure 1).

We then characterized each HML7 provirus’ context of integration, to assess its colocalization with cellular genes (Table 2). Among the 23 HML7 proviruses present in the human genome, 11 (48%) are integrated into human genes. Of these, all show an intronic localization and are found in antisense orientation with respect to the surrounding gene, except for one element (3q26.1) which results in the same direction of a gene producing a long intergenic non-protein coding RNA (LINC01322) (Table 2). As reported in the table, the majority of cellular genes colocalized with HML7 proviruses are known to be associated with human disorders.

### 3.3. Structural Characterization

**Figure 2:**
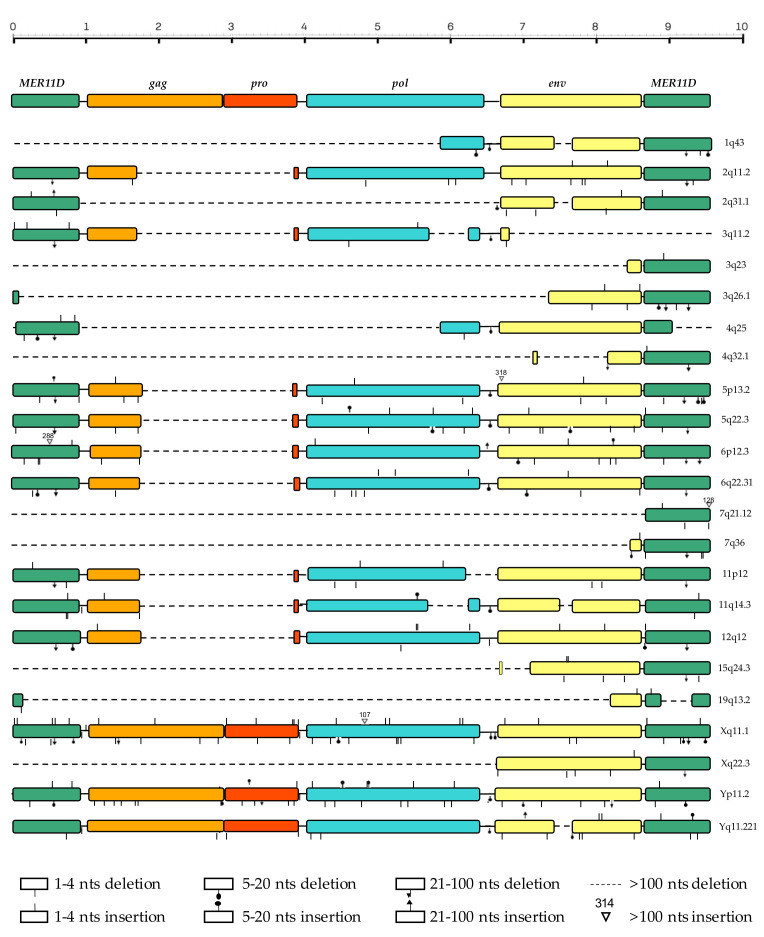
Structural characterization of HML7 proviral loci. The identified HML7 proviruses have been aligned with the Dfam proviral reference, and all insertions and deletions ≥1 nucleotide have been annotated.

The prototype sequence for the HML7 group, as assembled from the reference sequences for LTR (MER11D) and the internal portion (HERVK11D) present in the Dfam database, shows a typical proviral structure, in which 5′ LTR and 3′ LTR (897 bp each) flank *gag* (from nucleotide 1056 to 2924), *pro* (from nucleotide 2882 to 3929), *pol* (from nucleotide 4010 to 6487), and *env* (from nucleotide 6701 to 8647) genes. In order to characterize the structure of the individual HML7 proviruses, the 23 HML7 elements have been aligned with the reference sequence, as previously annotated with the positions of the single retroviral components, and all insertions and deletions have been marked (Figure 2). In general, the analysis revealed a defective proviral structure for the majority of HML7 members, which had a mean length of 4958 bases against the 9546 of the group reference. Accordingly, various HML7 proviruses lack one LTR (8 out of 23), and the majority is affected by extended deletions in one or more retroviral genes (Figure 2). Among these structurally incomplete sequences, 5 are particularly defective, with a length lower than 2000 bp due to the loss of around 2/3 of the proviral sequence at the 5′ side (Figure 2). A recurrent deletion of particular interest is the one affecting *gag* and *pro*: in fact, 9 HML7 proviruses showed the loss of a portion of ~2.200 bp spanning the two genes (nucleotides 1760–3970), while other 11 have lost the whole *gag*–*pro* genic regions (and, in some instances, also parts of the *pol* and *env* genes), leaving only 3 sequences with a complete (Xq11.1, Yp11.2) or almost complete (Yq11.221) proviral structure as compared to the Dfam reference (Figure 2). Besides this deletion, other minor deletions have found to be recurrent in some HML7 proviruses, including the loss of ~200 bases in *env* (nucleotides 7484–7684, 4 sequences) and 23 bases in the LTRs (nucleotides 595 to 617 in MER11D, deleted in 10 5′- and 14 3′-LTR sequences, respectively).

Given their defective structure, we assessed the residual presence of viral genes’ Open Reading Frames (ORFs) as well as recognizable functional domains in HML7 proviruses through RetroTector software [12] and the NCBI-conserved domains tool [34] (Table 3). As expected, Gag functional domains have been lost in the majority of sequences except for the proviruses at loci Xq11.1, Yq11.221, and Yp11.2, which show residual signatures typical of matrix (MA), capsid (CA), and nucleocapsid (NC) proteins. However, also the 9 HML7 sequences with the above *gag* recurrent deletion (nucleotides 1760–3970) still hold the 5′ portion of the gene, retaining accordingly the sole MA domain (Table 3). The *pro* gene is indeed the most defective, presenting predicted protease domain (PR) only in the most intact HML7 elements (Xq11.1, Yq11.221, Yp11.2). According to the highest integrity of the proviral 3′ portion, domains belonging to *pol* (reverse transcriptase—RT, Ribonuclease H—RNase H/RH, and integrase—IN) and *env* (surface—SU, and transmembrane—TM glycoproteins) are more represented, being recognized in the majority of HML7 proviruses (Table 3). However, the occurrence of multiple internal stop codons and frameshift in the ORFs of even the most intact HML7 proviruses suggest that these elements could have lost the ability to produce functional proteins, at least based on their nucleotide sequence in the reference genome assembly (Table 3). In addition, we assessed the presence of some structural features typical of class II betaretroviruses, which therefore have taxonomical value [35]. Particularly, HERV-K groups are known to have two Gag NC Zinc-fingers as well as a dUTPase domain at the N-terminal of Pro. Accordingly, these features have been identified in the three proviruses that retained the harboring genes (Table 3). Other important taxonomical markers are the presence of a nucleotide compositional bias (likely reflecting the ancestral action of encapsidated host RNA editing enzymes) and the translational strategy adopted to produce differing amounts of the various retroviral proteins (that can be based either on ribosomal frameshifting—fs, nonsense codon readthrough or splicing) [35]. Regarding nucleotide bias, HERV-K groups are known to present an increase in AT nucleotides, and HML7 proviruses showed accordingly an enrichment of the AT composition (around 60% of bases) (Table 3). Concerning translational strategy, retroviruses adopt splicing for the *env* gene transcripts, while *gag*–*pro* and *pro*–*pol* boundaries are usually translated using either ribosomal readthrough (fs = 0) or frameshifting (fs = −1 or +1). In the case of HERV-K betaretroviruses, the propensity is to have the *gag*, *pro,* and *pol* in different reading frames separated by “−1” frameshifts (“−1/−1” pattern). However, the HML7 proviruses that maintained these ORFs showed a different strategy, adopting instead readthrough for the *gag–pro* boundary (“0/−1”) (Table 3).

### 3.4. Phylogenetic Analyses

**Figure 3:**
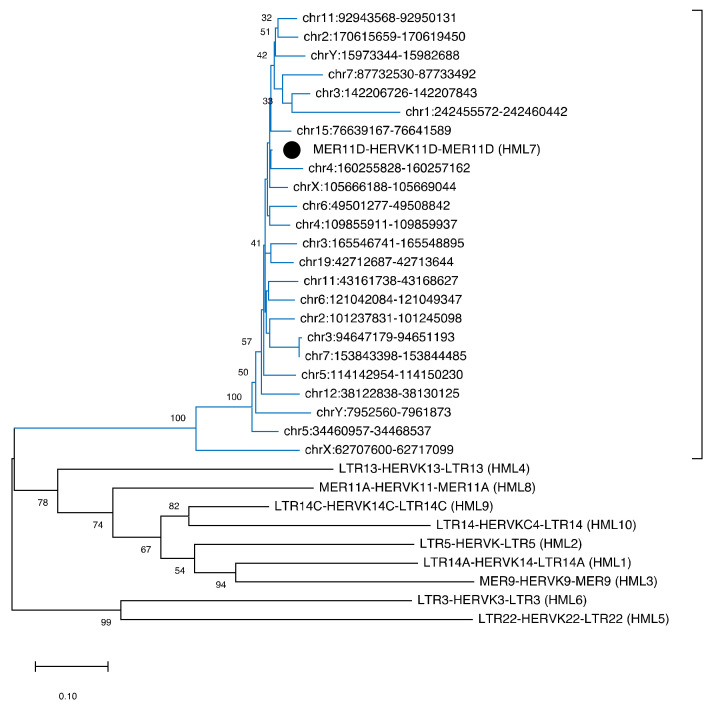
Phylogenetic tree of HML7 proviral loci. The identified HML7 proviruses were analyzed with the maximum likelihood method, including also the Dfam reference proviral sequences of all HERV-K groups (HML1 to HML10). Phylogenies have been statistically tested through the bootstrap method with 100 replicates. The monophyletic group formed by the HML7 proviruses and including the HML7 group Dfam reference (black dot) is highlighted with blue branches.

In order to characterize the phylogenetic relationships within the HML7 group as well as the ones with respect to the other HERV-K HMLs, we performed a series of analyses using the maximum likelihood (ML) and the neighbor-joining (NJ) methods. As shown in the ML tree (Figure 3), all the 23 HML7 proviruses form a unique monophyletic cluster, including also the group reference from Dfam, supported by the maximum bootstrap value. Even if some HML7 sequences were clearly more divergent as compared to the rest of members (e.g., the one in locus Xq11.1), the analysis did not reveal any supported subcluster within the main phylogenetic group (Figure 3). Additional NJ phylogenetic analyses carried out on individual retroviral portions, i.e., proviral LTRs and individual genes, confirmed the global homogeneity of HML7 members and the absence of internal clusters (Supplementary File S2). Similarly, also the NJ analysis of HML7 solitary LTRs gave the same result (data not shown).

### 3.5. Estimation of HML7 Loci Time of Integration and Comparative Genomics

**Figure 4:**
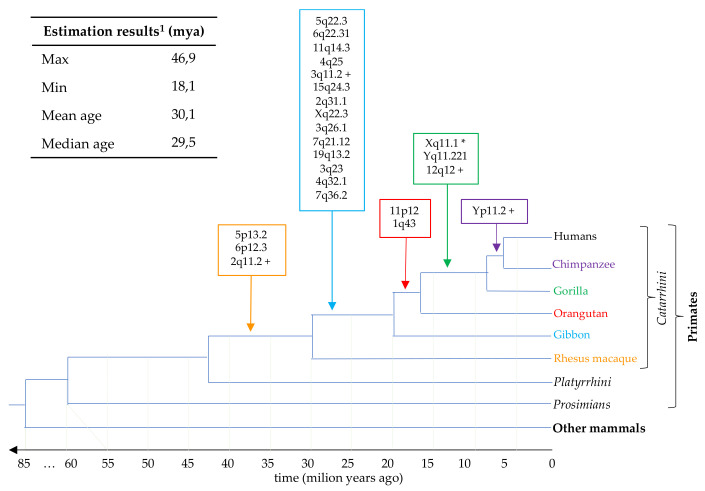
Time of integration of HML7 proviral loci in primate genomes. Temporal overview of the colonization of primate species by HML7 elements, based on the combination of time of integration estimation and comparative genomics analysis of each human locus in non-human primates. Each node indicates a speciation event, and the correspondent time is indicated in the line below. HML7 loci whose insertion occurred in centromeric or pericentromeric regions are marked with a * (Xq11.1) or a + (3q11.2, Yp11.2, 2q11.2 and 12q12), respectively, and their localization in non-human primates could possibly be affected by the lower comparability of constitutive heterochromatin. ^1^ Based on multiple approaches of divergence calculation; see materials and methods for further details.

Based on the retroviral replication cycle, it is known that proviral LTRs are identical at the time of integration, and then start to accumulate random mutations independently due to the normal effect of the host genome substitution rate (0.2% substitutions/nucleotide/million year in the case of humans). Indeed, the nucleotide divergence accumulated between the two LTRs of the same provirus is generally used to roughly estimate the time that had passed since their insertion in the host DNA. This approach has however some relevant limitations—such as the assumption that all genomic sites of integrations and retroviral portions are subjected to the same, neutral substitution rate—and obviously excludes from the analysis those proviruses that have lost one or both LTRs [13]. For this reason, we employed a multiple approach of age estimation that includes the traditional LTR vs. LTR divergence as well as the divergence calculated for each proviral portion (i.e., individual LTR and gag–pro, pol and env gene regions) with respect to a consensus sequence generated considering all group members. The latter should take into account all the independent random substitutions accumulated in the various HML7 elements, thus representing the putative ancestral HML7 proviral sequence. The final age of each HML7 locus has hence been expressed as the mean value obtained from the different estimations (Table 1). Overall, results showed that the majority of HML7 elements found in the human genome (*n* = 13) have been integrated in the primate lineage between 20 and 35 million years ago (mya), having hence been presumably acquired by rhesus macaque and gibbon prior to their evolutionary divergence to subsequent species (which occurred around 30 and 20 mya, respectively) (Figure 4). To corroborate this estimation, we used comparative genomics to identify for each HML7 locus the correspondent orthologous integration in non-human primates’ genome assemblies until the first species that acquired that element. Results confirmed that the transposition activity of HML7 group started in the rhesus genome (3 first integrations) and had its major expansion during gibbon speciation (30 to 20 mya, 14 first integrations), showing then occasional insertional events in orangutan and gorilla (2 and 3 acquired loci, respectively) (Figure 4).

## 4. Discussion

In the present study, the combination of RetroTector analysis and BLAT searches in genome assembly hg38 was used to identify the HERV-K(HML7) integrations present in human DNA, providing the first exhaustive description of this group. Results showed that the human genome harbors a total of 23 HML7 sequences, i.e., 9 more than the ones originally included in our first work of global HERV classification [5] (Table 1). This discrepancy is easily explained by the highly defective structure of these elements, leading to the loss of those retroviral motifs recognized by RetroTector during HERV identification and classification [11]. In addition, our analysis identified about 160 HML7 solitary LTRs, as the result of past recombination between the two LTR of the same provirus, leading to the removal of the inner genic portion (Supplementary File S1). Hence, the ratio between proviral sequences and solitary LTRs for HML7 group is around 1:7, in line with the assumption that LTR-LTR recombination process has been highly efficient in evolution, since ~90% of HERV insertions are currently represented by solitary LTRs [36]. A similar proportion (1:9) has been reported also for HML2 elements, the most characterized among HERV-K groups [16]. Interestingly, chi-square analysis revealed that HML7 proviral and solitary LTR integrations are not randomly distributed among human chromosomes, showing significant enrichment in chromosome X (*p* < 0.005) and depletion in chromosome 15 (*p* < 0.05). Moreover, a remarkable proportion of HML7 proviruses (17%) were found to be integrated in centromeric (Xq11.1) or pericentromeric (2q11.2, 3q11.2, 12q12) chromosomal regions. A similar localization has been reported for a subset of HERV-K(HML2) sequences that are found at multiple loci across the centromeres and pericentromeres of several chromosomes, as the result of human-specific amplifications [37,38]. The same authors analyzed the genomic landscape of centromeres in cancer, reporting a general reduction in centromeric DNA. Particularly, both solid and hematologic tumors showed marked alterations in the copy number of centromeric and pericentromeric repeats, including the above HML2 sequences that were hypothesized to drive possible gene conversion events observed at various pericentromeric loci [39]. Hence, in subsequent studies analyzing the possible effect of HML7 elements on the host, particular attention should be dedicated to HML7 centromeric and pericentromeric integrations, also in terms of possible impact on chromosomes’ evolution and stability in modern human populations.

The possible impact of HERVs on the host genome depends not only on their chromosomal position but also on the surrounding genic environment. Accordingly, we evaluated each HML7 locus context of integration, since proviral insertions in the vicinity or within human genes are potentially capable of modifying their expression (especially if they are in the same orientation) [7]. For example, HERV LTRs can provide alternative regulatory signals including promoters, enhancers, transcription factor binding sites and splicing donors/acceptors. However, antisense integrations can modify the surrounding gene activity as well; LTR promoters are in fact bidirectional and expressed HERV RNA can complementarily bind genic transcripts and form dsRNA, leading to their degradation. All these possible effects on cellular gene function acted as a selective pressure, with the propensity to maintain HERV insertions found in intergenic regions or, in the case of intra-genic insertions, occurring at non-translated intronic portions. Among the 23 HML7 proviruses identified, 11 (48%) are integrated within human genes and—as expected—almost all show intronic localization and antisense orientation with respect to them. An exception is represented by HML7 locus 3q26.1, which is integrated in the same orientation of a gene annotated to produce a long intergenic non-protein coding RNA (LINC01322) with unknown function (Table 2). Even if this HML7 locus is highly defective, retaining only a small portion of 5′LTR, a partial *env* gene and the 3′LTR, it would be interesting to assess if it takes part to the production of this non-coding RNA.

Structural characterization with respect to the Dfam proviral reference sequence (9546 nucleotides) revealed that the majority of HML7 elements show a defective proviral structure, with a mean length of 4969 bases and the frequent loss of one LTR (8 out of 23) and extended portions of one or more retroviral genes (Figure 2). A recurrent deletion of particular interest is the 2.200 bp one affecting *gag* and *pro*, found in 9 HML7 proviruses, while other 11 have lost the whole *gag*–*pro* portion. The fact that Gag polyproteins play a central role in virion assembly, release, and infectivity—being sufficient for the formation of virus-like particles—may suggest that this genic portion has been lost after the endogenization process, which favors intragenomic spread instead of extracellular replication, as already reported for *env* genes [12]. This would also explain the concomitant loss of *pro* gene, whose product was no longer needed to cleave Gag precursors into the major structural proteins MA, CA, and NC. Although generally more intact, HML7 *pol* and *env* genes are also often interested by deletion events, including the loss of ~200 nucleotides in *env* (7484–7684, 4 sequences). Similarly, the recurrent loss of the same 23 bases has been observed in both proviral LTRs (nucleotides 595 to 617 in MER11D, deleted in 10 5′- and 14 3′-LTR sequences, respectively).

Overall, only 3 HML7 members (13%) retained a complete (Xq11.1, Yp11.2) or almost complete (Yq11.221) proviral structure. The fact that all were integrated in sexual chromosomes allows to speculate that they may have been subjected to less recombination and substitution events as compared to the other HML7 proviruses, maintaining a higher structural integrity. This would be in line with the fact that—in species with genetic sex-determination—sexual chromosomes have evolved non-recombining regions in which recombination has repeatedly become suppressed [40].

As expected from their general defective structure, the great majority of HML7 proviruses lost most of the functional domains normally predicted in retroviral sequences and, among the one with highest integrity as identified by RetroTector, none present intact ORFs due to the introduction of internal stop codons and frameshifts (Table 3). However, one should consider that these predictions were based on the proviral sequences identified in human genome assembly; it is hence possible that individual human genomes still hold HML7 loci with residual coding potential, deserving further investigations in transcriptomic data [41]. Concerning typical Class II structural features, as in the other HERV-K groups, HML7 elements also present two Gag NC Zinc-fingers as well as a dUTPase domain at the N-terminal of Pro. As expected, the compositional bias reported previously for the other HML groups [12,42] was confirmed also in HML7 proviral genomes, which shared an enrichment in AT content (around 60% of bases) and a consequent decrease in C and especially G nucleotides (22% and 18% in average, respectively) (Table 3). While it has been demonstrated that co-packaging of APOBEC cellular cytidine deaminase gives a bias for A in HIV genome—and probably acted on the genomes of several retrotransposons as well—the mechanisms behind the T bias are not so clear. The fact that the evolution of APOBEC3 antiviral defensive systems has been driven by the diffusion of ERV elements in mammalian genomes [43] make possible, however, the idea that ancestral retroviral elements were threatened by additional genome editing systems, leading to different compositional biases. HML7 elements indeed represented an exception in terms of translational strategy adopted to produce the needed amounts of the various retroviral proteins. In fact, HERV-K betaretroviruses show the propensity to have the *gag*, *pro,* and *pol* in different reading frames separated by “−1” frameshifts (“−1/−1” pattern) [12], while the HML7 proviruses that maintained these ORFs adopted readthrough for the *gag–pro* boundary (“0/−1”) (Table 3).

Phylogenetic analysis showed that HML7 proviruses as well as solitary LTRs form a unique monophyletic cluster, clearly divided from other HML groups and supported by the maximum bootstrap value. Despite such an absence of internal clusters, some HML7 sequences showed a divergent nucleotide sequence with respect to the other group members, which was particularly evident in locus Xq11.1, i.e., the only centromeric HML7 provirus (Table 1, Figure 3). Its highest divergence may indeed be linked to this localization at the chromosome X centromere. In fact, if on the one side sexual chromosomes are associated with a low recombination rate (as mentioned above), on the other side, rapid evolution is a known characteristic of repeated centromeric DNA that could have acted on this specific HML7 provirus as well, making it particularly divergent as compared to the others [44]. The same pressure could have led even to the loss of this HML7 element in some primate species, as observed in gorilla genome assembly (Table 1). In line with this general action dependent on its genomic localization, the same phylogenetic distance is found by analyzing the individual proviral portions (Supplementary File S2) and is thus unlikely to be due to single events of recombination or secondary insertions.

Concerning time of integration estimation, the traditional approach based on the divergence between the two LTRs of the same provirus was coupled with the comparison of each LTR and proviral gene with respect to a consensus sequence, allowing the inclusion, in the analysis, of not only the elements retaining both LTRs (8 out of 23), but also the other HML7 loci. The only HML7 element excluded from age estimation was the centromeric Xq11.1 locus, which gave unreliable results due to its divergent nucleotide structure. Results indicated the main period of HML7 diffusion to be comprised between 20 and 35 mya (13 elements, Figure 4). To corroborate this estimation, we relied on the comparative localization of each HML7 locus orthologue in non-human primates’ genome assemblies, until the first species that acquired that element (i.e., the oldest common ancestor, Table 1). Results confirmed that the transposition activity of the HML7 group started in the rhesus genome (3 first integrations) and had its major expansion during gibbon speciation (30 to 20 mya, 14 first integrations) (Figure 4). Of note, the presence of later occasional HML7 first insertions in the orangutan (2 loci) and gorilla (3 loci) genomes likely suggest and extended residual activity of the group (Figure 4).

## 5. Conclusions

Overall, the comprehensive characterization of the HML7 group composition, localization, and dynamics of diffusion in primates here presented provides for the first time an exhaustive genomic description of this poorly investigated group. This opens the field to the study of individual HML7 elements’ specific variation within human population, their residual transcriptional activity in the different tissues and—by consequence—their eventual modulation in diseased conditions, to unveil unknown contributions of the group to human evolution and physiopathology [41]. The latter can be multifaceted and are however the result of complex interactions with the different tissues/cellular systems rather than on a general mechanism of HERV activation. As a mere example, the HML7 locus 2q11.2 was found to be upregulated in human PBMC following innate immunity stimulation by a mimicked bacterial infection [45], while no modulation was observed in the presence of viral infections (HIV [46] and SARS-CoV-2, Grandi et al., unpublished data) or autoimmunity (multiple sclerosis—Grandi et al., unpublished data). This and many other pieces of evidence suggest that HERV modulation is a complex phenomenon, influenced by multiple factors, and cannot be understood based on the general upregulation of a certain group in a given condition; thus the field indeed asks for dedicated high-throughput expression studies to definitively assess these elements’ impact on our health.

## Figures and Tables

**Table 1:** HML7 proviral sequences identified in the human genome assembly GRCh38/hg38.

Locus ^1^	Strand	Coordinates	Age (Milion Years)	o.c.a ^2^	Reference
1q43	+	242457056–242460436	22.9	orangutan	Vargiu et al.
2q11.2 ^P^	+	101237831–101245098	32.3	rhesus	Vargiu et al.
2q31.1	−	170615659–170619450	24.3	gibbon	this study
3q11.2 ^P^	+	94647178–94651193	46.9	gibbon	Vargiu et al.
3q23	+	142206726–142207843	29.9	gibbon	this study
3q26.1	+	165546697–165548895	37.0	gibbon	this study
4q25	−	109855914–109859920	27.9	gibbon	Vargiu et al.
4q32.1	−	160255828–160257162	22.0	gibbon	this study
5p13.2	−	34460957–34468537	29.8	rhesus *	Vargiu et al.
5q22.3	−	114142954–114150230	29.3	gibbon	Vargiu et al.
6p12.3	−	49501277–49508842	29.3	rhesus *	Vargiu et al.
6q22.31	+	121042084–121049347	26.6	gibbon	Vargiu et al.
7q21.12	−	87732402–87733417	40.0	gibbon	this study
7q36.2	−	153843398–153844485	31.0	gibbon	this study
11p12	−	43161738–43168627	18.1	orangutan (gorilla *)	Vargiu et al.
11q14.3	−	92943568–92950131	26.4	gibbon	Vargiu et al.
12q12 ^P^	+	38122838–38130125	36.4	gorilla	Vargiu et al.
15q24.3	+	76639167–76641589	24.3	gibbon	this study
19q13.2	−	42712688–42713786	30.5	gibbon	this study
Xq11.1 ^C^	−	62707600–62717099	-	gorilla °	Vargiu et al.
Xq22.3	−	105666188–105669044	22.0	gibbon	this study
Yq11.221	−	15973344–15982688	38.2	gorilla *	Vargiu et al.
Yp11.2	−	7952560–7961873	38.2	chimp	Vargiu et al.

^1^ HML7 elements integrated in pericentromeric and centromeric regions are indicated with a ^P^ and a ^C^, respectively. ^2^ Oldest Common Ancestor: HML7 loci converted into a solitary LTR during primate speciation (*) or lacking an orthologue in intermediate primate species (°) are also indicated.

**Table 2:** HML7 proviral sequences colocalized with cellular genes.

HML7 Locus	Colocalized Gene Info				
	Name	Portion	Description	Function	Associated Diseases
1q43 (+)	PLD5 (−)	intronic, antisense	Phospholipase D family member 5	Hydrolyzes phosphatidylcholine	Type 7 nephrotic syndrome; hemopneumothorax
2q31.1 (−)	MYO3B (+)	intronic, antisense	Myosin IIIB	Probable actin-based ATPase with protein kinase activity. Required for normal cochlear development and hearing	Autosomal recessive deafness 30; entropion
3q23 (+)	GK5 (−)	intronic, antisense	Glycerol kinase 5	Glycerol degradation, triacylglycerol biosynthesis	Type 1 Diabetes Mellitus 3 and 7
3q26.1 (+)	LINC01322 (+)	intronic, sense	long intergenic non-coding RNA 1322	-	-
4q25 (−)	LRIT3 (+)	intronic, antisense	Leucine rich repeat Ig-Like transmembrane domains 3	May regulate fibroblast growth factor receptors and affect their post-translational modification	Congenital stationary night blindness
5q22.3 (−)	KCNN2 (+)	intronic, antisense	Potassium calcium-activated channel subfamily N member 2	Forms a voltage-independent potassium channel activated by intracellular calcium following membrane hyperpolarization	Lingual-facial-buccal dyskinesia and aceruloplasminemia
6p12.3 (−)	GLYATL3 (+)	intronic, antisense	Glycine-N-acyltransferase like 3	Catalyzes the conjugation of long-chain fatty acyl-CoA thioester and glycine, an intermediate in primary fatty acid biosynthesis	-
7q21.12 (−)	RUNDC3B (+)	intronic, antisense	RUN domain-containing protein 3B	Encodes a predicted RAP2-interacting protein. May play a role in RAS-like GTPase signaling pathways	-
7q36.2 (−)	DPP6 (+)	intronic, antisense	dipeptidyl peptidase like 6, transcript variant 6	Member of S9B family of serine proteases (without detectable activity). Promotes cell surface expression of KCND2 potassium channel and modulates its gating activity	Autosomal dominant mental retardation; paroxysmal familial ventricular fibrillation
15q24.3 (+)	SCAPER (−)	intronic, antisense	S-phase cyclin A associated protein in the endoplasmic reticulum	Cyclin A/Cdk2 regulatory protein that transiently maintains cyclin A in the cytoplasm	Intellectual developmental disorder and retinitis pigmentosa; brachydactyly
Xq22.3 (−)	IL1RAPL2 (+)	intronic, antisense	Interleukin 1 receptor accessory protein like 2	Orphan receptor in the IL1R superfamily	Cinca syndrome; Muckle-Wells syndrome

The table shows in order: The locus of each HML7 and its strand, the name of the colocalized gene and its strand, the intronic/exonic and sense/antisense localization of the HML7 element, and the description of the gene product and its function. In the last column, the pathologies associated so far with each gene are also reported (source: OMIM database).

**Table 3:** Coding potential and functional domain predicted for the HML7 proviral loci.

	gag						pro				pol						env				Translation		GC%
HML7 Locus	Shift	Stop	MA	CA	NC	NC ZnF	Shift	Stop	PR	dUTPase	Shift	Stop	RT	RH	IN ZnB	IN core	Shift	Stop	SU gp	TM hr	gag/pro Shift	pro/pol Shift	
**1q43**											6	7			x	x	1	1	x	x			39.6
**2q11.2**			x								3	1	x	x	x	x	5	5	x	x			40.6
2q31.1															x	x			x	x			38.8
**3q11.2**			x								6	2		x	x								41.1
3q23																			x	x			41.9
3q26.1																							39.0
**4q25**											6	8					1	5	x	x			39.2
4q32.1													x	x		x							40.6
**5p13.2**			x								4	5	x	x	x		1	1	x	x			40.3
**5q22.3**			x								8	4	x	x	x	x	9	1		x			40.2
**6p12.3**			x								0	6	x		x	x	8	1					40.0
**6q22.31**			x								4	8	x	x	x	x	4	4	x	x			40.1
7q21.12																							43.0
7q36.2																							43.4
11p12			x										x	x					x	x			40.1
**11q14.3**			x								1	1	x	x	x		1	5		x			40.3
**12q12**			x								3	7	x	x	x	x	2	2	x	x			39.3
15q24.3																			x	x			38.8
19q13.2																				x			40.3
**Xq11.1**	1	11	x	x	x	xx	2	4	x	x	8	4	x	x	x	x	4	5	x	x	0	−1	40.0
Xq22.3																							39.7
**Yq11.221**	3	7	x	x	x	xx	0	3	x	x	3	7	x	x	x	x	6	5	x	x	0	−1	39.6
**Yp11.2**	0	3	x	x	x	xx	2	2	x	x	11	8	x		x	x	7	7	x	x	0	0	40.0

The table reports the presence of the different retroviral open reading frames and the occurrence of internal stop codonsand frameshifts in the 14 most intact HML7 loci, as identified by RetroTector software (locus name in bold). For each HML7 provirus, the presence of functional domains and taxonomical signatures was also predicted and is indicated with the symbol “x.” Abbreviations not explained in the main text: ZnF = Zinc finger motif, ZnB = Zinc-binding domain, gp = glycoprotein, hr = heptad repeats, GC% = percentage of GC nucleotides.

## Data Availability

All the data presented in this study are included in the article. HML7 genomic sequences analyzed can be visualized on the human reference genome through the correspondent coordinates in USCS Genome Browser (https://genome.ucsc.edu accessed on 30 March 2021).

## Supplementary Materials

The following are available online at https://www.mdpi.com/article/10.3390/biology10050439/s1, Supplementary File S1: HML7 solitary LTR sequences identified in the human genome assembly GRCh38/hg38, Supplementary File S2: Neighbor joining phylogenetic analyses of individual HML7 proviral portions.
