# Supplementary material for: HERV-K(HML7) Integrations in the Human Genome: Comprehensive Characterization and Comparative Analysis in Non-Human Primates
Source: Biology (Basel). 2021 May 14;10(5):439. doi: 10.3390/biology10050439 (PMC8156875; doi:10.3390/biology10050439)
Supplement: Supplementary file 1 [file biology-10-00439-s001.zip › Supplementary_file_S1.pdf]

Supplementary file S1. HML7 solitary LTR sequences identified in the human genome assembly GRCh38/hg38

chromosome	start	end	strand
chr1	9124260	9125061	+
chr1	12705236	12706103	+
chr1	46756512	46757382	+
chr1	49801979	49802860	+
chr1	59921250	59922156	+
chr1	70751600	70752473	+
chr1	89018103	89018958	+
chr1	91466416	91467282	+
chr1	101555484	101556347	+
chr1	102789849	102790716	-
chr1	119786221	119786997	-
chr1	147103237	147104108	+
chr1	194415875	194416748	+
chr1	221196566	221197440	-
chr1	223303543	223304412	-
chr1	248882051	248882924	-
chr2	116288174	116289070	+
chr2	123112737	123113595	-
chr2	139912644	139913516	-
chr2	186375135	186376029	+
chr2	208482219	208483079	-
chr2	210430307	210431185	+
chr2	228714598	228715472	-
chr3	6306935	6307809	-
chr3	20921429	20922302	-
chr3	35173081	35173952	+
chr3	55248733	55249674	-
chr3	84896402	84897275	-
chr3	106360640	106361513	-
chr3	151808620	151809492	-
chr3	165676473	165677333	-
chr3	191267466	191268349	-
chr4	54408991	54409888	-
chr4	58817735	58818677	+
chr4	75954591	75955464	+
chr4	76281767	76282659	-
chr4	79591919	79592822	-
chr4	87291010	87291906	-
chr4	92217751	92218615	+
chr4	97968745	97969555	+
chr4	98479300	98480167	+

chr4	99231610	99232473	+
chr4	132308349	132309221	-
chr4	135863957	135864827	-
chr4	144542550	144543432	+
chr4	160364606	160365479	+
chr4	175594684	175595581	+
chr5	20179960	20180854	+
chr5	34421366	34422239	-
chr5	42834271	42835144	-
chr5	42931194	42931678	+
chr5	79706001	79706874	-
chr5	80470850	80471722	+
chr5	145442437	145443284	-
chr5	152358180	152359046	+
chr5	154072994	154073884	-
chr5	155811614	155812510	-
chr5	164544075	164544925	+
chr5	176945381	176946277	+
chr6	65689809	65690680	-
chr6	66482360	66483256	-
chr6	73459395	73460248	+
chr6	81440742	81441615	+
chr6	85485885	85486778	-
chr6	145133108	145133989	+
chr6	157900407	157901278	-
chr7	2428388	2429259	-
chr7	30509034	30509894	-
chr7	37584110	37584982	-
chr7	64143949	64144837	-
chr7	78832666	78833538	+
chr7	89545124	89545997	-
chr7	93869864	93870757	-
chr7	97597744	97598618	+
chr7	100718143	100719043	+
chr7	137205564	137206459	+
chr7	141962353	141963243	+
chr7	153244679	153245552	-
chr8	5591426	5592257	+
chr8	13767314	13768182	-
chr8	29541987	29542887	+
chr8	39618139	39619008	-
chr8	46019600	46020470	+
chr8	54089877	54090774	+
chr8	124965592	124966462	+
chr8	134288114	134289034	+
chr8	134378504	134379372	-

chr9	3110324	3111220	-
chr9	104394521	104395392	-
chr9	111591784	111592657	+
chr9	118116845	118117718	-
chr9	122809057	122809945	+
chr10	37007972	37008851	-
chr10	45175393	45176261	-
chr10	51580893	51581765	-
chr10	53517584	53518450	+
chr10	92912284	92913158	-
chr10	94939164	94940060	-
chr11	26316119	26316989	-
chr11	37135432	37136317	+
chr11	38784968	38785851	+
chr11	67681787	67682660	-
chr11	100031208	100032063	+
chr12	34110212	34111112	+
chr12	55255985	55256861	+
chr12	74259974	74260871	+
chr12	87578803	87579676	-
chr12	125499323	125500131	+
chr12	125974130	125975029	+
chr13	22983460	22984463	-
chr13	24646276	24647145	+
chr13	58241108	58241973	+
chr13	95430298	95431194	-
chr14	23944073	23944952	-
chr14	47529907	47530771	+
chr14	70539162	70540034	+
chr14	82285575	82286448	+
chr14	93809245	93810143	-
chr14	96421919	96422792	-
chr14	106732833	106733722	+
chr16	65813156	65814052	+
chr17	7168880	7169774	+
chr17	27181224	27182097	-
chr17	35341379	35342251	+
chr17	41341831	41342685	-
chr17	44340426	44341299	+
chr17	53748065	53748937	+
chr17	78082075	78082947	-
chr18	14796667	14797496	-
chr18	14809000	14809821	-
chr18	64144672	64145568	+
chr19	9763869	9764752	+
chr19	20692433	20693313	+

chr19	34797632	34798228	-
chr19	36736030	36736895	+
chr21	13426517	13427330	+
chr22	21071693	21072588	+
chr22	36203145	36203924	-
chr22	42498284	42499175	-
chrX	6355226	6356116	-
chrX	6567091	6567987	-
chrX	8153787	8154681	+
chrX	14848131	14849029	+
chrX	36662040	36662920	+
chrX	43028039	43028913	-
chrX	58152151	58153049	-
chrX	58444869	58445755	-
chrX	75571978	75572791	+
chrX	76010642	76011509	+
chrX	76822154	76823051	-
chrX	80205526	80206102	-
chrX	81560681	81561553	+
chrX	90854488	90855358	+
chrX	135023852	135024722	+
chrX	149552503	149553378	-
chrX	149926119	149926994	+
chrY	4436932	4437804	+
chrY	19058771	19059642	+
chrY	22110338	22111231	-
chrY	22475175	22476068	+
