# Supplementary material for: HERV-K(HML7) Integrations in the Human Genome: Comprehensive Characterization and Comparative Analysis in Non-Human Primates
Source: Biology (Basel). 2021 May 14;10(5):439. doi: 10.3390/biology10050439 (PMC8156875; doi:10.3390/biology10050439)
Supplement: Supplementary file 1 [file biology-10-00439-s001.zip › Supplementary_file_S2.pdf]

Supplementary file S2. Neighbor joining phylogenetic analyses of individual HML7 proviral portions:

a) provirus-associated LTRs

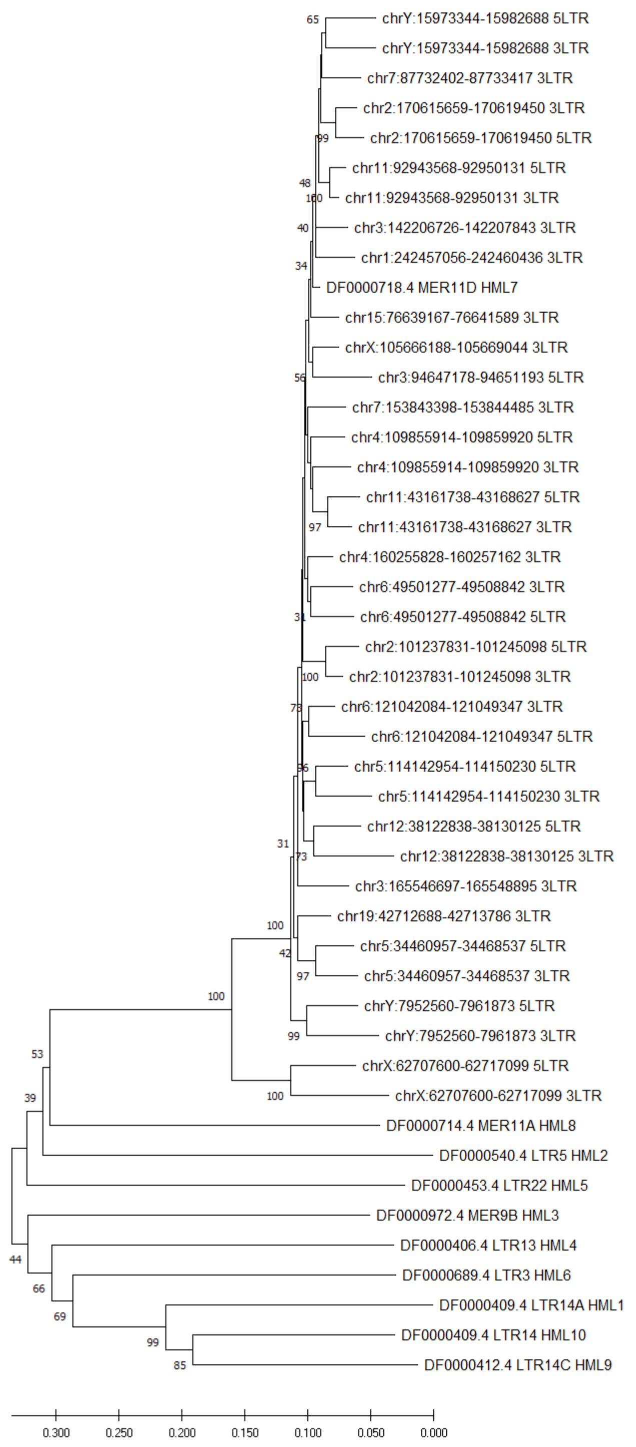

b) *gag-pro* genes

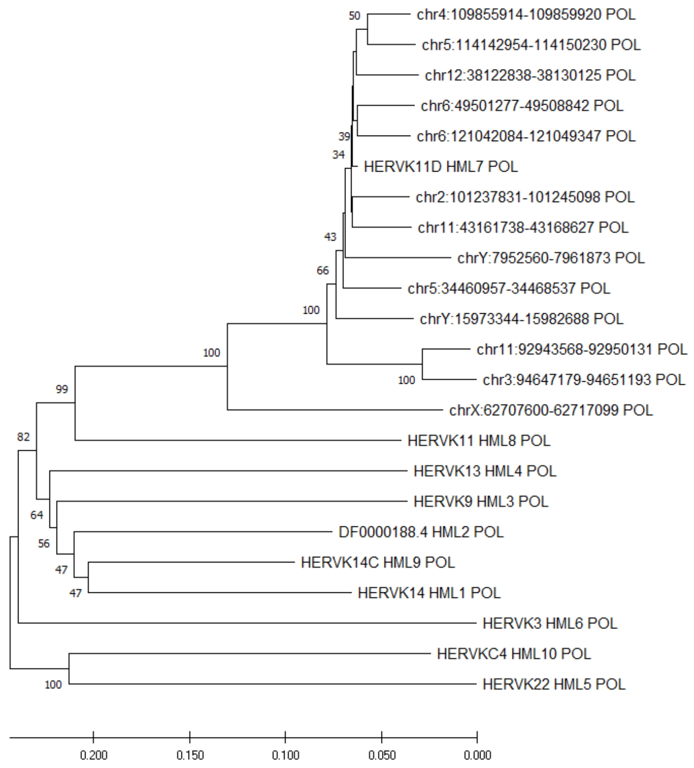

c) *pol* gene

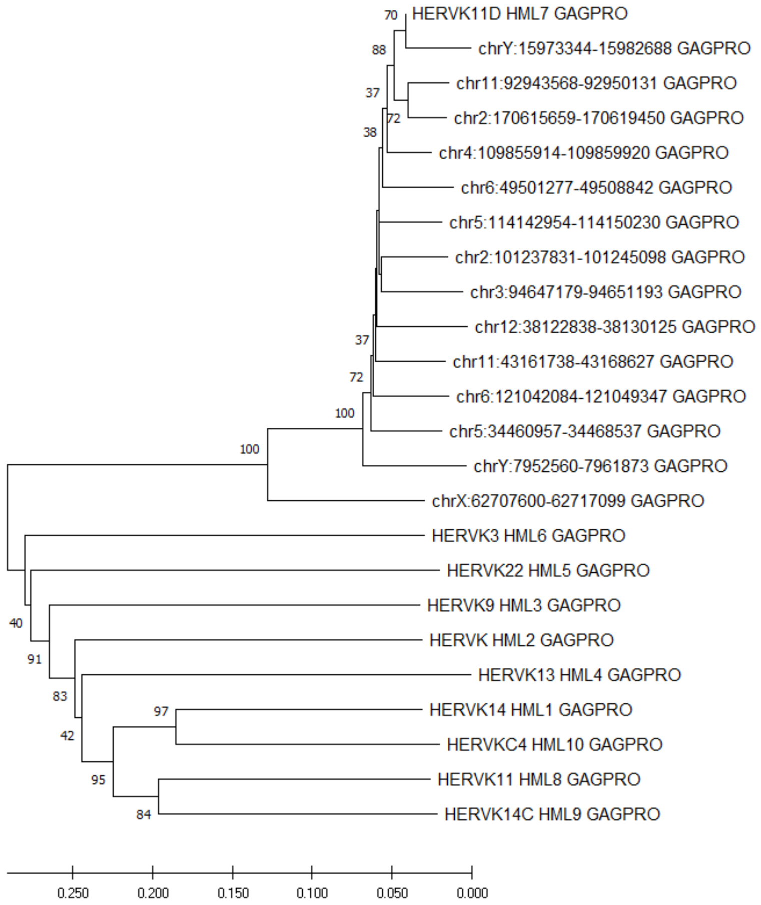

d) *env* gene

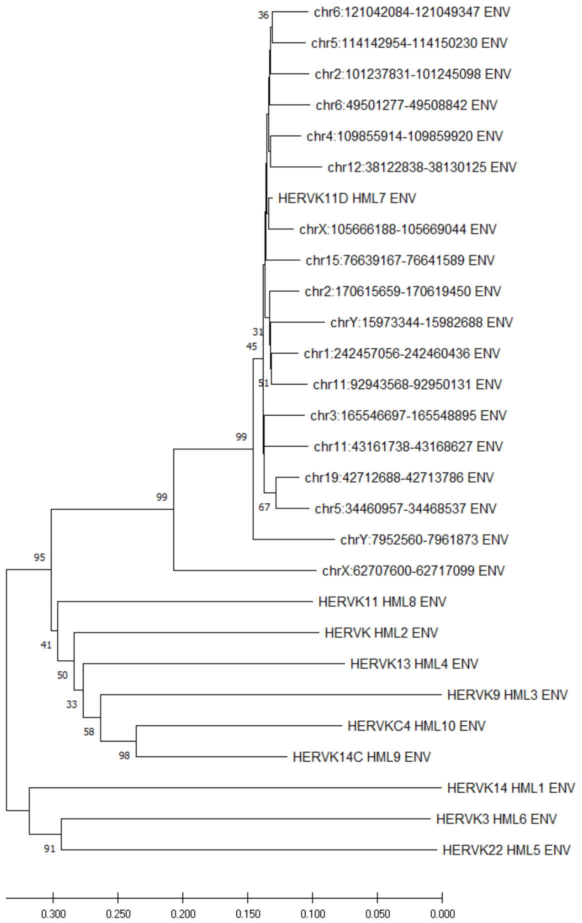

chr6:121042084-121049347 ENV
chr5:114142954-114150230 ENV
chr2:101237831-101245098 ENV
chr6:49501277-49508842 ENV
chr4:109855914-109859920 ENV
chr12:38122838-38130125 ENV
HERVK11D HML7 ENV
chrX:105666188-105669044 ENV
chr15:76639167-76641589 ENV
chr2:170615659-170619450 ENV
chrY:15973344-15982688 ENV
chr1:242457056-242460436 ENV
chr11:92943568-92950131 ENV
chr3:165546697-165548895 ENV
chr11:43161738-43168627 ENV
chr19:42712688-42713786 ENV
chr5:34460957-34468537 ENV
chrY:7952560-7961873 ENV
chrX:62707600-62717099 ENV
HERVK11 HML8 ENV
HERVK HML2 ENV
HERVK13 HML4 ENV
HERVK9 HML3 ENV
HERVKC4 HML10 ENV
HERVK14C HML9 ENV
HERVK14 HML1 ENV
HERVK3 HML6 ENV
HERVK22 HML5 ENV
36
31
45
51
99
99
67
95
41
50
33
58
98
91
0.300
0.250
0.200
0.150
0.100
0.050
0.000
